# EXTENDING THE FAMILY TREE: NEW FACES OF DEMENTIA CAREGIVERS

**DOI:** 10.1093/geroni/igae098.1315

**Published:** 2024-12-31

**Authors:** Jyoti Savla, Karen Roberto

**Affiliations:** Chair; Co-Chair

## Abstract

Research has predominantly focused on spouses and adult children as primary caregivers for individuals living with dementia. However, evolving family dynamics necessitate a critical examination of caregivers beyond this immediate circle. This symposium explores the understudied area of informal dementia caregiving, which includes care provided by extended family members, stepchildren, and chosen kin. This emergent focus is crucial in redefining the family caregiver archetype amid changing family structures. McConnell & Haggar employ social network analysis to underscore variations in family/non-kin participation in care networks, influenced by factors such as disease severity and caregiver availability. Roberto & Savla provide an in-depth exploration of historical and current family dynamics that shape niece and nephew caregivers’ responsibilities, practices, and outcomes. Savla & Roberto address the individual and family challenges faced by older adult sibling caregivers, as well as identify factors that facilitate and impede their caregiving capacities. Silverstein et al. leverage longitudinal data to explore the impact of kinship strength and early relationship histories on stepchildren’s support for stepparents living with dementia. Flatt et al. synthesize insights from their multiple studies on the health outcomes of sexual and gender minority dementia caregivers within biological and chosen familial networks. Collectively, these studies underscore the complexity and diversity of caregiver roles within families, emphasizing the need for supportive programs and policies tailored to their unique circumstances. Findings serve as a call for action to recognize and support the wide range of dementia caregivers in today’s society who demonstrate remarkable resilience in navigating dementia care challenges.

